# Differences in Electronic Consultation Conversion Rates Between Advanced Practice Providers and Board-Certified Dermatologists

**DOI:** 10.2196/83922

**Published:** 2026-01-30

**Authors:** Dakota Hitchcock, Sabrina Newman

**Affiliations:** 1 Department of Internal Medicine, Saint Joseph Hospital, 1960 Ogden Street Suite 400, Denver, CO, 80218, United States, 1 303-318-1540; 2 Department of Dermatology, University of Colorado School of Medicine, Department of Dermatology, Aurora, CO, United States

**Keywords:** e-consult, telehealth, dermatology, advanced practice provider, teledermatology

## Abstract

In this analysis of dermatology e-consults at a large academic health system, advanced practice providers had nearly threefold higher conversion rates to in-person visits compared to board-certified dermatologists, with potential implications for access and resource utilization.

## Introduction

Electronic consultations (e-consults) have become an increasingly valuable tool in improving access to specialty care, reducing unnecessary in-person referrals, and supporting timely management of patients by primary care providers [1,2]. By allowing clinicians to consult with specialists asynchronously through the electronic health record, e-consults can help streamline workflows, decrease wait times, and conserve specialist resources [2,3]. Dermatology services receive a high number of e-consult requests, likely due to the visual diagnostic nature of the specialty [3,4]. As the use of e-consults expands across health care systems, understanding how different provider types use this tool, particularly in high-demand specialties such as dermatology, is critical to optimizing efficiency and effectiveness. Furthermore, identifying whether variations in conversion patterns reflect provider-level practice differences or system-level routing processes is essential for ensuring that e-consults function as intended.

## Methods

We conducted a retrospective analysis to evaluate whether e-consult conversion rates differed by provider type, specifically comparing advanced practice providers (APPs), including nurse practitioners and physician assistants, to board-certified dermatologists. e-consult data specific to dermatology were extracted from the University of Colorado Hospital electronic health record system for the period of January 2020 to April 2025. An e-consult was considered “converted” if it resulted in a subsequent in-person specialist visit or full referral, rather than being resolved entirely through asynchronous communication.

In this system, e-consults are routed to APPs versus dermatologists primarily based on provider availability rather than consult content or patient acuity. As a result, patients evaluated by APPs and physicians likely represent comparable clinical populations, reducing the likelihood that differences in conversion rates were driven by systematic triage of more complex cases to one provider group.

## Results

A total of 2572 dermatology e-consults were submitted during the study period. Of these, 1205 were addressed by APPs, with 321 (26.6%) resulting in conversion to an in-person visit (Table 1). In contrast, only 125 of the 1367 e-consults addressed by physicians (9.1%) were converted (Table 2). e-consults managed by APPs were nearly three times more likely to lead to an in-person referral compared to those managed by physicians.

**Table 1.** Total number and percentage of e-consults converted from advanced practice professionals.

e-consult converted	N (%)
No	884 (73.4)
Yes	321 (26.6)
Total	1205 (100.0)

**Table 2.** Total number and percentage of e-consults converted from dermatologists.

e-consult converted	N (%)
No	1242 (90.9)
Yes	125 (9.1)
Total	1367 (100.0)

## Discussion

This analysis reveals a notable difference in e-consult conversion rates between APPs and physicians. This disparity suggests potential differences in how each provider group approaches triage and decision-making in specialty care. If APP-handled e-consults were converted at the same rate as physician-handled e-consults, over 200 additional dermatology clinic appointments during the study period may have been available for patients with higher-acuity needs. Despite this variation in appointment conversion, it is important to note that the majority of e-consults from both groups were resolved without the need for in-person follow-up. This reinforces the broader value of e-consults in improving efficiency and reducing unnecessary specialist visits and aligns with current literature [2,3].

The higher conversion rate observed among APPs may reflect a range of underlying factors. One possibility is that APPs may be more likely to convert e-consults conservatively due to comparatively less specialty-specific training or comfort managing complex cases. Importantly, in our system, APPs and dermatologists receive e-consults based largely on provider availability rather than clinical complexity. This reduces the likelihood that differences in patient or case characteristics explain the observed variation. Existing literature on provider-level differences in e-consult use and impact have shown mixed results. For example, one study comparing e-consults submitted by nurse practitioners and family physicians found that nurse practitioners were more likely to report that the consultation led to new clinical guidance and less likely to say it avoided an in-person referral [5]. In contrast, a systematic review of referral practices found no significant difference in overall referral rates between nurse practitioners and family physicians [6]. However, these studies largely examine differences among referring providers rather than responding providers. Because our study investigates variation among the providers performing the e-consults themselves, it represents a novel contribution to the literature. To our knowledge, no published studies have specifically examined provider-level variation in dermatology e-consult outcomes from the specialist side, underscoring the importance of our findings.

While our findings shed light on differences in provider behavior, they also raise questions about the clinical appropriateness of these conversions. Without detailed outcome data, it remains unclear whether the higher conversion rate among APPs were clinically necessary or reflective of a lower threshold for referral. Future research should explore the clinical drivers and downstream outcomes of converted e-consults, considering patient complexity, consult content, and specialty-specific considerations.

In addition to clinical impact, the higher conversion rate among APPs may have broader implications for system efficiency and resource use. Given the higher conversion rate, APP-managed e-consults may increase health care utilization, with potential cost implications for patients and health systems. Assuming a standard new patient visit billed at a level 3 or level 4 (estimated reimbursement US $120–$180 per visit), the additional ~200 appointments potentially consumed due to higher APP conversion rates translates to an estimated US $24,000–$36,000 in additional health care costs during the study period. Future work could further investigate whether these conversions lead to improved outcomes or represent avoidable costs.

This study contributes to the growing body of literature on e-consult optimization and provider practice variation. As health systems increasingly adopt team-based models of care and integrate APPs more fully into specialty workflows, ensuring consistent and effective use of e-consults across provider types will be essential. Implementing structured guidance, standardized triage protocols, and targeted training modules, particularly for APPs, may help promote more consistent decision-making and appropriate referral thresholds. Additionally, health systems may consider establishing limitations or clinical guidelines regarding the types of dermatologic conditions appropriate for independent APP e-consult management to ensure high-quality care, reduce unnecessary referrals, and minimize avoidable health care costs. By equipping all members of the care team with the tools and guidance needed to manage e-consults effectively, we can improve access, preserve specialist capacity, and enhance the overall efficiency of care delivery.

## Abbreviations

APP: advanced practice provider
E-consults: electronic consultations

## References

[1] Vimalananda VG, Orlander JD, Afable MK (2020). Electronic consultations (E-consults) and their outcomes: a systematic review. J Am Med Inform Assoc.

[2] Vimalananda VG, Gupte G, Seraj SM (2015). Electronic consultations (e-consults) to improve access to specialty care: a systematic review and narrative synthesis. J Telemed Telecare.

[3] Keely E, Liddy C, Afkham A (2013). Utilization, benefits, and impact of an e-consultation service across diverse specialties and primary care providers. Telemed J E Health.

[4] Bradley C, Smith L, Youens K, White BAA, Couchman G (2023). Formalizing the curbside: digitally enhancing access to specialty care. Proc (Bayl Univ Med Cent).

[5] Liddy C, Deri Armstrong C, McKellips F, Keely E (2016). A comparison of referral patterns to a multispecialty eConsultation service between nurse practitioners and family physicians: The case for eConsult. J Am Assoc Nurse Pract.

[6] Laurant M, Reeves D, Hermens R, Braspenning J, Grol R, Sibbald B (2005). Substitution of doctors by nurses in primary care. Cochrane Database Syst Rev.

